# Supplementary material for: Are carbon emissions trading and green financial instruments synergistic? -Comprehensive quantitative research based on content analysis
Source: PLoS One. 2024 Mar 7;19(3):e0298601. doi: 10.1371/journal.pone.0298601 (PMC10919601; doi:10.1371/journal.pone.0298601)
Supplement: S1 Data — (ZIP) [file pone.0298601.s001.zip › Data/地级市Z_F工作报告.zip!/╣π╢1⁄2/╓╨╔╜╩╨/└┤╫╘╣1⁄2╓┌║┼í╛┬φ┐╦╩2╛▌═°í┐_2011.docx]

各位代表：

我代表市人民政府向大会作政府工作报告，请予审议，并请政协各位委员和其他列席人员提出意见。

“十一五”时期及2010年工作回顾

“十一五”时期，是我市发展历程中极不平凡的五年，也是我市经济健康发展、社会和谐进步、人民群众得到更多实惠的五年。市政府在省委、省政府和市委的正确领导下，在市人大及其常委会和市政协的监督支持下，坚持以邓小平理论和“三个代表”重要思想为指导，深入贯彻落实科学发展观，全面实施《珠江三角洲地区改革发展规划纲要》，凝心聚力调结构、保增长、促发展、惠民生，大力推进经济强市、和谐中山、宜居城市、法治社会、文化名城和社会主义新农村建设，经济社会发展取得新成就，胜利完成2010年和“十一五”各项目标任务，在科学发展道路上迈开新步伐。

有效应对国际金融危机，经济综合实力显著增强。2010年，全市生产总值1826亿元，增长13.5%；人均生产总值跨越1万美元大关；工业总产值5309.6亿元，增长20.4%；地方财政一般预算收入139.4亿元，增长26.2%；全社会固定资产投资660.4亿元，增长21%；出口总值225.1亿美元，增长26.9%；社会消费品零售总额648.1亿元，增长18.2%。与“十五”期末比，生产总值、工业总产值、地方财政一般预算收入、全社会固定资产投资均实现翻番增长。企业经济效益稳步提高，2010年规模以上工业企业利润比“十五”期末增长193%。

调整优化产业结构，经济发展方式加快转变。三次产业结构由2005年3.5∶60.9∶35.6转变为2010年2.8∶58.7∶38.5，产业结构趋向高级化和适度重型化。拥有国家级产业基地27个，省级科技专业镇15个，省级以上名牌名标397个，年产值百亿级企业2家。规模以上轻重工业增加值结构由61.9∶38.1转变为59.8∶40.2。2010年，高新技术产品产值占工业总产值31%，新能源产业产值增长27.3%。传统产业升级加快，传统支柱产业工业产值年均增长17.8%。服务业稳健发展，金融业取得突破性成绩，新增16家金融机构，12家企业国内外上市，上市融资比“十五”时期翻一番。旅游业总收入年均增长13.5%。内外源经济结构进一步调整，外贸依存度从188%下降到112%。农业现代化步伐加快，农业亩产值1.24万元，居全省前列，农业总产值比“十五”期末增长20.8%。科技创新能力不断提升，五年累计投入19.6亿元用于科技创新，带动企业研发投入80.1亿元。拥有国家级示范生产力促进中心和企业技术中心3家、省级工程中心和企业技术中心62家，行业集群联盟标准18项，发明专利授权量比“十五”时期增长5.3倍。电动车辆国家工程实验室进驻中山，兆瓦级风电叶片、大功率永磁同步电机等取得关键技术突破。连续四届获评全国科技进步考核先进市。

加强基础设施建设，城市功能日臻完善。财政五年累计投入106.7亿元推进城市基础设施建设，实现较大幅度增长。“五横六纵七高速九加密”公路主干线稳步推进，完成古神公路一期、城桂路二期、南北外环路、博爱路及起湾道改造、长江路大桥和康华路大桥、广珠西线二期、广珠城轨等一批重大交通项目，公路通车里程累计1917公里，公路密度达106.5公里/百平方公里。大力推进广佛江珠城轨、广东西部沿海铁路、南沙港铁路（含中山港铁路）等跨界重大工程前期工作，积极争取省支持将深中跨海大桥建设项目列入“十二五”规划。全市形成“一港五区”港口布局。能源、电源、电网及信息化建设加快，完成嘉明电厂扩建及500千伏香山、桂山输变电工程等。实施中顺大围达标加固、西河水闸重建等工程，提前一年完成省城乡水利防灾减灾工程建设任务。文化艺术中心、博览中心、中山职业技术学院、中山纪念中学扩建、市一中初中部迁建、疾病预防控制中心、中医院、博爱医院儿科大楼、第二人民医院一期、新泰安市场等一批城市设施投入使用。“三旧”改造实现当年1万亩目标。

统筹城乡协调发展，城镇化建设取得新成就。推进城乡规划建设、基础设施、产业布局、社会管理、公共服务一体化。市财政五年累计投入211.2亿元用于社会事业和公共服务，城市管理水平进一步提高。超过80%农村从业人员从事二三产业，城乡居民收入比缩小为1.71∶1，城镇化率为86.3%，达到全省较好水平。实现城乡公路公交、电信宽带、广播电视、自来水、卫生站、农家书屋和健身园全覆盖。新农村建设加快，完成农村股份合作制和村级统一核算改革，稳步推进“村改居”，建成59个农民集中居住小区。农业基础设施建设加强，建成“硬底化”主干农路581公里、高标准农田3.4万亩、现代农业园区8万亩。政策性农业保险加快推进。2010年全市村集体经济平均年收入突破1千万元。

深入推进改革开放，经济社会管理增添活力。创新行政审批方式改革成效明显，取消市级设定审批事项，全面推行“一网式”审批。市级政府机构改革基本完成，事业单位人事制度改革取得阶段性成果。开展城乡金融服务一体化综合改革试点和省科技金融结合试点工作。整合市属企业投融资平台，推行代建制等投融资新模式。创新财政管理体制，对欠发达镇实施“一级财政”加“专项补助”政策。实现工业服务业用水、污水处理、生活垃圾处理同价。在全省率先推行流动人口积分制管理。积极参与珠三角一体化，牵头实现珠中江机动车年票互认和班车公交化。实施产业和劳动力“双转移”，合作建成4个省级产业转移园，劳动力转移工作获评全省第一。外经贸转型步伐加快，一般贸易出口占全市出口比重由“十五”期末29.5%上升到37.3%，机电产品和高新技术产品出口占比分别达64.9%和28.6%。累计189家来料加工企业实现不停产转型，2010年出口转内销总额增长33.1%。

大力保护生态环境，绿色中山建设成效明显。成为首批国家级生态建设示范区，所有镇成为全国环境优美乡镇。与“十五”期末相比，单位生产总值能耗下降18%，二氧化硫和化学需氧量排放量下降13.5%、35.5%，主要污染物总量减排和珠江综合整治考核名列全省前茅。建成20个污水处理厂，城镇生活污水处理率达87.5%。中心组团、北部组团垃圾综合处理基地投产运营，中心城区生活垃圾无害化处理率近100%。整治内河涌1227公里，主要河道水质明显改善。建设五桂山生态保护区，完成林分改造5.3万亩，森林覆盖率达19.1%，成为省林业生态市。绿道中山段全线贯通。

积极发展民生事业，和谐社会建设跃上新台阶。全面推进十大民生工程，人民生活持续改善。2010年城镇居民年人均可支配收入和农村居民人均年纯收入分别为25357元和14928元，富裕程度进一步提高。城乡居民储蓄余额1443.7亿元，是“十五”期末2倍，居民消费价格指数涨幅控制在3%以内。大力促进就业创业，2010年新增就业8万人，高校毕业生就业率达97%，城镇登记失业率控制在2.1%，创建国家级创业型城市通过省初检。推进城乡一体的社会保险体系建设，城镇职工社会保险总参保668.6万人次，基本实现城乡居民基本养老保险、住院和门诊基本医疗保险全覆盖。推进住房保障，建成廉租房527套、经济适用房1000户，完成1852户低保危房改造。完善社会救助体系，慈善事业发展加快。“十一五”投入127.4亿元优先发展教育，所有镇区成为省教育强镇。实现城乡免费义务教育，基础教育均衡优质发展稳步推进，高等教育普及化，职业教育超常规发展，民办教育规范发展，建设五桂山职教园区，建成万人技师学院。医疗卫生综合服务能力不断提升，国家级卫生镇达11个。人口自然增长率5.2‰，居民人均期望寿命达78.2岁。文化名城建设加快，公共文化设施覆盖城乡，所有镇区文化站达省特级标准，5个项目纳入国家历史文化遗产代表名录。全民健身运动广泛开展，竞技体育成绩显著。文明城市创建有新进步。获评全国双拥模范城。圆满完成漩口援建任务。

加强政府自身建设，民主法治进程加快。坚持依法治市，自觉接受市人大的依法监督和市政协的民主监督。坚持重大工作向市委、市人大报告和向市政协通报制度，五年办理人大代表议案建议582件，政协提案1023件，答复率为100%。行政复议委员会试点经验在全国推广，规范行政处罚自由裁量权工作取得明显成效。开展政务公开和网络问政，推进行政效能创新，政府执行力不断提高。连续3年行政经费零增长。健全市长接待日等信访制度，群众来信来访办结率达92%。设立人民调解组织490个，261个社区（村）建立综治信访维稳工作站。完成“五五”普法，法律援助工作扎实有效。安全生产持续平稳，平安中山建设成效突出，社会大局和谐稳定。

“十一五”时期，外事侨务、港澳事务、台务、民族宗教、审计、统计、国防建设、妇女儿童、老龄、残疾人、气象、人防、档案、方志等各项工作在原有基础上取得较大进步，为中山经济社会发展作出重要贡献。

各位代表！回顾“十一五”，我们深刻体会到：必须始终坚持狠抓发展，以经济建设为中心，以平稳较快发展应对各种严峻考验，不断增强可持续发展的实力。必须始终坚持转型升级，以市场为导向，通过政策调控，加快转变经济发展方式，提高产业竞争力和自主创新能力，不断促进经济上规模增效益。必须始终坚持以人为本，大力发展社会民生事业，做到发展为了人民、发展依靠人民、发展成果由人民共建共享，努力增进人民群众幸福感，切实维护社会稳定，不断促进社会和谐。必须始终坚持改革开放，创新体制机制，推进重点领域和关键环节改革，焕发全市上下干事创业的活力。充分发挥伟人故里和侨乡优势，不断提高对外开放水平和质量。必须始终坚持求真务实，认真学习实践科学发展观，准确把握内外环境变化，一切从实际出发，探索独具中山特色的科学发展之路。必须始终坚持领潮争先，发扬中山敢为天下先的优良传统和时代精神，敢于迎接挑战，勇于克服困难，善于化危为机，不断提高城市竞争力和综合实力。“十一五”时期的成功实践，加深了我们对发展规律的认识，夯实了推动科学发展的基础。

回顾“十一五”，我们倍感取得的成绩来之不易，积累的经验弥足珍贵，创造的精神财富影响深远。这是省委、省政府和市委正确领导的结果，是市人大及其常委会、市政协及各民主党派、无党派人士监督支持的结果，是全市干部群众团结奋斗的结果，是海内外各界关心帮助的结果。在此，我代表市政府，向全市广大工人、农民、知识分子、企业家、干部群众，向各民主党派、工商联、各人民团体、无党派人士和社会各界人士，向驻我市解放军和武警官兵、公安民警，向所有关心支持中山发展的老领导、老同志、港澳台同胞、海外侨胞和国内外友人表示衷心感谢！

回顾“十一五”，我们清醒认识到，我市经济社会发展还存在一些突出问题。纵向比虽然成绩显著，横向比与标兵差距在拉大，与追兵距离在缩小。对照省的要求与兄弟地市先进经验，我们产业结构调整亟需提速，经济发展方式转变有待加快；转型升级任重道远，发展内生动力亟待增强；人才瓶颈亟待破解，科技创新能力仍显不足；资源环境压力日趋加大，发展空间严重受限；影响稳定因素增多，社会管理难度增大；昂扬奋进的精神状态仍需增强，行政执行力有待进一步提升；发展中积累的一些深层次矛盾和问题亟待解决处理等。我们要高度重视面临的问题，攻坚克难，迎难而上，采取更加积极的措施切实予以解决。

“十二五”展望及2011年工作安排

“十二五”时期，是我市加快转变经济发展方式、实现科学发展新跨越的关键五年，挑战与机遇并存，压力与动力共在。综观国内外形势，经济全球化深入发展，我国处于可以大有作为的重要战略机遇期。我省区域合作发展全面推进，我市“十一五”积累的实力与经验为持续发展奠定了坚实基础。与此同时，国际经济格局深度调整，国家宏观经济形势复杂，我省改革发展深层次问题不断凸显，兄弟城市争先恐后竞争态势日趋激烈，我市加快发展任务艰巨、挑战巨大。我们要积极抢抓机遇，勇敢迎接挑战，敢于直面竞争，更好地肩负起推进科学发展、增进民生福祉的历史重任。未来五年，我们要正确处理经济发展中好与快的关系，好字当头、好中求快；正确处理管理体制中统与分的关系，宜统则统、宜分则分；正确处理发展取向中人与物的关系，以人为本、民生为重；正确处理增长动力中内与外的关系，内外并举、协调推进；正确处理产业升级中新与旧的关系，新旧并重、协同发展；正确处理统筹发展中城与乡的关系，城增优势、乡显特色。

“十二五”时期政府工作的指导思想是：高举中国特色社会主义伟大旗帜，以邓小平理论和“三个代表”重要思想为指导，深入贯彻落实科学发展观，以科学发展为主题，以加快转变经济发展方式为主线，围绕“加快转型升级、建设幸福中山”这个核心，积极实施珠三角《规划纲要》，增强人均意识与资源环境意识，大力完善基础设施，提升城市功能，调整经济结构，优化生态环境，强化民生保障，抓紧实施人才强市战略，不断提高市民文化素养和文明素质，不断提升人民群众幸福感，努力构筑经济发达、社会和谐、法治进步、文化繁荣、环境优美、人民安康、城乡一体的和美家园，加快建设适宜创业、适宜创新、适宜居住的全面协调可持续发展的新型城市。

“十二五”时期的发展目标是：到2015年，全市人均生产总值提前五年实现比2000年翻两番目标，经济发展方式转变取得显著进展，城乡面貌有新变化，社会软实力显著提升，民生福祉显著改善，科学发展的体制机制日益完善。

——综合实力跃上新台阶。经济保持平稳较快发展，全市生产总值年均增长11%，人均生产总值年均增长10%，到2015年全市人均生产总值超过10万元。产业结构更加优化，服务业增加值占生产总值比重达45%，消费、投资、出口协调拉动。自主创新能力不断提高，研究和试验经费支出占生产总值比重达2.5%。为建设幸福中山夯实物质基础。

——社会发展取得新成效。社会事业全面进步，城乡一体社会保障体系更加完善，城镇户籍从业人员养老保险参保率达99%左右，人口自然增长率不超过7‰；就业更加充分，城镇登记失业率控制在3.5%以内；国民教育体系日臻完善，高中阶段毛入学率达100%左右，高等教育毛入学率达60%；公共服务体系更加健全，民主法治建设全面加强，构建和谐社会取得新进展。

——民生福祉达到新水平。人民生活更加殷实，城镇居民人均可支配收入年均增长8%，农村居民人均纯收入年均增长9%，城乡收入比缩小到1.62∶1。人民生活质量和水平不断提高。

——环境质量实现新提升。生态环境明显改善，资源利用效率显著提高。单位生产总值能耗五年累计下降18%，主要污染物排放总量显著减少，森林覆盖率达19.4%以上，耕地保有量不少于4.968万公顷，低碳经济、循环经济加快发展。

2011年是我市站在新的历史起点上承前启后、继往开来的重要一年，做好今年工作意义重大。我们要增强加快发展的使命感和紧迫感，凝聚全市人民的智慧和力量，围绕抓转型、促发展、优布局、强交通、惠民生的工作重点，励精图治，发奋图强，努力实现“十二五”良好开局，开创科学发展新局面。

2011年经济社会发展的主要预期目标是：生产总值增长11%，单位生产总值能耗下降4.1%，二氧化硫、化学需氧量、氨氮、氮氧化物排放量达到国家和省控制指标，全社会固定资产投资增长14%，社会消费品零售总额增长14%，外贸出口总值增长10%，地方财政一般预算收入增长12%，城镇居民年人均可支配收入增长8%，农村居民人均年纯收入增长9%，居民消费价格总指数升幅在4%左右，城镇登记失业率控制在3%以内，人口自然增长率控制在7‰以下。力争经济较快增长，超额完成目标任务，为长期平稳较快发展奠定坚实基础。

实现上述目标，今年必须做好以下十项工作。

一、加快产业结构调整，推进现代产业体系建设

改造提升优势传统产业。实施传统产业振兴、专业镇升级、产业集群壮大“三大战略”。编制实施专业镇“一镇一策”提升规划，重点促进灯饰光源、家用电器、五金制品、纺织服装等产业集群升级，推动产业集群向营销、研发等高端环节延伸，打造千亿级产业集群。创建国家创新型现代照明产业集群试点和国家创新型科技园区。做优名牌名标，培育一批传统产业龙头企业。

做强做优先进制造业。坚持走新型工业化道路，工业增加值增长12%。重点发展船舶制造和海洋工程、电梯装备、大型铸锻件、机械制造等装备制造业。加快建设临港装备制造业基地，推进中铁南方装备制造基地、中船造船基地等20个重点项目建设并尽早投产，促进先进制造业集聚发展。引进大型优质先进制造企业和项目，培育成长性好的本土企业，做强配套型企业。

推进战略性新兴产业跨越式发展。加强新兴产业规划引导和政策扶持，重点发展新能源、半导体照明、新型电子信息、健康医药等新兴产业。推进明阳海上风电产业基地、盟固利锂电池、北汽福田新能源商用汽车、纬创液晶光电、航空天然气发动机国家级测试基地等项目，建设好广东省新能源产业基地、广东省新光源高新技术应用基地、中国电子中山基地、国家健康科技产业基地和华南现代中医药城。力争新兴产业增加值占生产总值比重达4.2%。

加快发展现代服务业。促进服务业发展提速和结构优化，提高第三产业在三次产业中的比重，提高现代服务业在服务业中的比重，服务业增加值增长12%。安排市服务业重点项目29个，计划年度投资65亿元。重点发展金融、现代物流、商贸会展、工业设计、综合旅游等服务业。建设东区中心商务区、西区服务业综合改革试验区、广东软件和数字家庭产业孵化基地等服务业集聚区。推进普洛斯、雨润等物流项目，创建综合保税物流区。加快建设南朗海上温泉、神湾盛世游艇会等项目，推动旅游业大发展。加快制造业服务环节分离发展服务业，加强服务业统计。

积极发展现代农业。财政“三农”投入8.8亿元，促进农业增效、农村发展、农民增收。加快建设“一带两区十基地”农业现代园区，南方绿博园申报国家级现代农业园区。以古神公路带为重点，建设规模化产业化绿化苗木基地。建设“硬底化”主干农路195公里和高标准基本农田6000亩，加快危桥改造。培育农业名牌产品，发展绿色食品、无公害农产品，提高粮食综合生产能力。完善基层农业技术推广体系，加强农产品质量监管和动植物疫病防控。

二、加快打造新增长点，提高经济发展质量效益

实施“三个一百”战略。强化政策导向，坚持项目带动，加快转型升级。推动100家外商投资企业及来料加工企业转型升级，支持100家内资企业做强做大，引进100家优质企业。推进20个广东现代产业500强项目建设，培育发展现代产业100强。发展总部经济，建设总部集聚区。增强民营经济活力，推动个体工商户转型升级。实行企业就地转型升级与异地转移发展相结合，推进产业和劳动力“双转移”取得新突破。

提高资源使用效益。增强人均意识、亩产意识，提高资源投入产出比，科学统筹项目、用地、资金等资源利用。产业发展资金增加到4亿元，重点发展用地少、效益高、创新多、结构优、能耗低、生态好项目。建立全市土地统筹管理制度，强化用地准入门槛，盘活闲置低效用地，创新土地征用模式，用地向重大基础设施和优质项目倾斜。加强城轨站场、新建干线公路沿线土地综合开发。加快石岐综合试验区、华南家电产业服务创新区等建设，年内实施“三旧”改造3万亩。推进质量强市活动。

加强科技创新。加快人才、技术等高端要素集聚，构建企业为主体、市场为导向、产业为依托、政产学研结合的创新体系。实施创新型企业孵化工程，力争高新技术产品产值占规模以上工业总产值比重达35%。打造一批省级以上工程中心和企业技术中心，引进一批行业共性技术平台，创建国家重点实验室，加强重点领域科技攻关。加快建设科技金融结合示范市。设立1500万元人才发展专项资金，实施领军人才培养、优秀企业家培育、专业技术人才创新能力提升、高技能人才振兴工程，加强科技产业园、博士后工作站、留学生创业园等载体建设。鼓励企业做大做强品牌，推动中山制造向中山创造转变。

强化金融服务。推进城乡金融服务一体化综合改革试点工作，构建多元化金融体系。引进境内外金融机构，发展村镇银行、小额贷款公司、融资性担保公司等本土金融机构，启动中山农信联社改制商业银行工作，加快企业上市进程。加强政银企合作，强化中小企业融资扶持，推进政策性农业保险和出口信用保险。发挥中科中山股权投资基金、科技创新投资基金作用，发展创业投资和风险投资。大力推进投融资体制改革，积极推行代建制、BT、BOT等模式，解决建设资金紧缺问题。

建设发展新平台。启动“一个平台两大基地”计划。积极争取省与国家支持，整合孙中山故里和东部沿海区域资源，与大型央企合作，规划建设新的战略平台，努力将其打造成为转型升级先导区、民生福祉示范区、社会建设试验区。推动风电装备基地和电动汽车产业基地跨越发展，打造新能源产业集聚区。

三、加快内外协调拉动，增强经济内生动力

加强重点领域投资。扩大投资规模，优化投资结构。安排市“十二五”重点项目69项，总投资1287亿元。今年安排市重点项目79项，年度计划投资196.8亿元；省重点项目67项，年度计划投资86亿元。健全重点项目推进机制，加强重点项目储备管理。积极申报省重点项目，争取国家和省专项资金。激活民间投资，鼓励多种投资主体参与基础设施、市政公用等领域投资。

提高招商引资质量。统筹全市招商引资管理，建立重大招商引资项目库。实施项目准入制度，着重引进高端领域、功能契合、产业配套项目。积极参加省年初向中央企业、年中向民营企业100强、下半年向世界500强企业三场重大招商活动。强化服务业招商，力争服务业实际吸收外资占吸收外资总额30%。提高招商队伍专业化水平，发挥商会行业协会作用，加强与知名中介机构招商合作。提高项目落地率并促其尽快产生效益。

大力开拓市场。推动进口与出口、吸收外资与对外投资并重，深度开发香港、美国、欧盟等传统市场，拓展东盟、印度、中东、拉美等新兴市场。培育本市跨国经营主体，鼓励建立自主出口品牌。强力开拓国内市场，形成“中山家居”等特色产品合力，组织“中山产品全国行”。促进加工贸易转型升级，支持企业创立内销品牌和国内营销体系，加快非法人资格来料加工企业原地不停产转型。加大重要原材料、先进技术设备、关键零部件和一般贸易进口。加强“大通关”建设。

扩大消费需求。发展消费新增长点，开拓绿色、健康、文化、信贷、家庭服务等新型消费模式。繁荣商业平台，推进兴中广场、富华、益华等商圈建设，加快轨道站场商贸功能开发。做好家电下乡、以旧换新等工作，发掘农村消费潜力。控制物价过快上涨，规范市场经济秩序，改善消费环境。

四、加快提升城市功能，增创城市发展新优势

优化城市发展格局。走发展集聚、产业提升、城市拓展相协调的新型城市化道路，打造“一主三副多节点”城市发展新格局，进一步发挥火炬区集聚、辐射、带动效益。适度拉大城市框架，提高主城区首位度，把主城区打造为交通路网畅通、服务功能完备、高端要素集聚、辐射带动力强的首善之区。扶持欠发达镇加快发展，促进市域协调发展。推进国民经济与社会发展规划、城市总体规划、土地利用规划有效对接，全面提升城市管理水平。

构筑现代综合交通运输体系。建设珠三角三大经济圈交通枢纽，提升城市区位优势。实施“交通先行再先行、公交优先再优先”的基础设施发展战略，以路为纲改善交通基础设施，着力缓解交通拥堵问题，力争一年初见成效、三年大见成效、五年达到畅行目标。加快建设“五横六纵七高速九加密”干线路网，加强与珠三角机场、港口、轨道等重大设施对接，积极配合推进深中跨海大桥、江珠高速北延线及江门至番禺高速、中开高速、广佛江珠城轨、中莞城轨、广东西部沿海铁路、南沙港铁路（含中山港铁路）等规划建设。加快市际市域道路对接，落实“3015”工程。新岐江公路、新十水线古镇小榄段、城桂公路一期路面改造、横二线阜港公路改造等年内投入使用，加快105国道中山段改造和中环路、广珠中线二期、东部快线等建设，新开工建设古神公路二期、小榄快线、横二线东段、中山港大桥扩建等项目。完善绿道网和城市慢行交通系统。

推进功能项目建设。加强项目科学论证，加快500千伏和220千伏各等级输变电工程、嘉明电厂三期、粤海集团热电联产、市域天然气利用、黄圃港和莲员路片区改造等建设，启动与建设好市一中高中部、市第二人民医院二期等一批教育卫生重点工程。推进长江水库增容、蓄淡抗咸水源保护、南朗中心二河河道整治及堤防建设、三乡排涝等工程。

建设社会主义新农村。落实镇村规划，优化村庄布局，实施综合整治，促进村庄建设整齐化、村民居住集聚化、环境卫生干净化、村容村貌美丽化。规范农民自建房管理，推进宅基地换房，引导农民有序集中居住。做好名镇名村建设工作，推进8个新农村示范点建设。培育新型农民，倡导现代乡村文明。认真做好村居换届工作。

五、加快发展生态文明，建设环境友好社会

打造绿色环境。大幅增加市镇两级绿化投入，充分调动社会力量，大力推广全民义务植树，营造爱绿植绿护绿风尚，实施城区、镇区交通主干道绿化提升工程。推进景观林、公共绿地和沿河沿路绿化带建设，改造紫马岭、逸仙湖公园，启动田心森林公园项目。加强岐江河亲水宜居长廊、古神公路绿色生态带和东部沿海红树林湿地公园保护预控，构建“一山一河一带一园”生态保育格局。完成7500亩林分改造、23条村“万村绿”建设任务。

坚持低碳发展。开展全国生态文明建设试点，构建资源节约和环境友好的生产消费方式。完善节能目标考评责任制，抓好工业、交通、建筑等重点领域节能。推广合同能源管理，建设资源能源节约示范工程。落实清洁生产企业分级管理，建立再生资源回收体系。实施固定资产投资项目节能评估审查，严格执行主要污染物排放总量前置审核制度，实现排放总量和浓度“双控制”。

加强环保建设。强化重点污染源监管和农业面源污染防治，建设北部组团垃圾处理中心二期、南部组团垃圾处理中心、固体废物综合处理中心。实施全市污水处理“一盘棋”，继续推进污水处理厂及管网建设，加快雨污分流。保护水源地环境，确保饮用水安全。加强生态综合治理，做好五桂山生态保护区、长江水库水源涵养区等生态敏感区保护工作。

六、加快改革攻坚步伐，积极发展区域合作

促进统筹城乡综合改革。合理界定市镇管理权限，更加注重市级统筹，更加注重调动镇区积极性。推进简政强镇事权改革，强化镇级社会管理和公共服务职能。完善农村股份合作制和村级统一核算改革，加快“村改居”，推动条件成熟的农村股份合作经济组织转型为现代企业。鼓励土地承包经营权流转，发展规模经营，完善农村集体经济管理模式。

推动社会管理改革。创新社会管理服务模式，构建政府与社会功能互补、协调互动的社会管理网络。规范发展社会组织，鼓励其参与社会管理服务。完善政府购买服务机制，促进公共服务供给多元化，探索建立镇区服务中心、社区服务代办点。大力发展社会服务事业，推广社区工作和志愿服务。完善流动人口积分制管理。

深化公共财政管理体制改革。优化财政支出结构，重点投向基础设施、产业升级和民生领域。完善预算编制执行管理，深化国库集中支付改革，建立绩效主导的财政分配机制，稳妥推进财政预算公开。完善转移支付和市镇分税分成体制，规范一级财政管理。加强专项资金管理，提高财政资金放大效益。依法治税理财，加强税收征管，确保应收尽收。规范非税收入管理。

强化国有资产管理体制改革。加强市属国有资产监管，确保国有资产保值增值增活力。整合市属国有企业，明确主导产业，完善企业产权结构和法人治理机制，鼓励非公有制企业参与国有企业改革。

加强区域合作。落实珠三角《规划纲要》“四年大发展”目标，主动承接中心城市辐射，积极融入珠三角一体化，打造沿海产业带先进制造业基地和珠江口西岸重要服务业基地。推进珠中江经济圈通信同城化、供水同网化等工程，深化交通、能源、环保、公共服务合作。加强与穗深港澳在技术、金融、物流、创意、人才、服务外包等领域合作，积极与环珠三角经济带合作发展。做好“双到”扶贫开发工作。

七、加快改善民生保障，发展基本公共服务

增加城乡居民收入。坚持富民与强市同步、居民收入增长与经济发展同步、劳动报酬增长与劳动生产率提高同步。健全职工工资正常增长长效机制，提高职工福利水平，增加农村劳动力工资性收入，促进居民财产性收入增长，扩大中等收入群体比重。

促进就业创业。市财政安排6000万元推进就业创业。健全就业培训、咨询、融资等服务体系，年内城镇新增就业5万人。优化扶持创业政策体系，争创国家级创业型城市。构建市、镇、高校三级人力资源市场网络，加强社区劳动保障服务站功能建设，推动高校毕业生就业创业。完善大嫂工作坊、社区车间、农业工作坊等平台，帮扶重点人员、困难群体就业再就业。加强劳务合作，保障企业用工需求。

完善社会保障。市财政投入3.8亿元用于社会保障，推进社会保险城乡一体化。提升城乡养老保险保障水平，逐步提高医疗保险待遇，社会基本医疗保险参保率达95%以上。加强保障性住房供给，建立低保家庭危房改造长效机制。做好民政工作，安排8500万元用于最低生活保障等社会救助，改善低收入家庭和困难群体生活质量。加强红十字会和慈善总会建设，推进残疾人事业。

八、加快发展各项事业，促进社会全面进步

促进社会和谐。构建适应社会转型的利益协调、诉求表达、矛盾调处、权益保障机制。建立工资保障金制度，落实最低工资标准，推进工资集体协商。加强企业人文关怀，构建和谐劳动关系。深化平安中山建设，积极化解历史遗留问题。发挥镇区综治信访维稳中心作用，加强社会治安综合治理，切实维护社会稳定。不断强化法制权威，严厉打击各种犯罪。加强安全生产，建立消防交通等安全监管长效机制。做好应急管理，有效处置突发事件。

推进教育现代化。在前些年高强度投入教育设施基础上，进一步强化质量强教、内涵提升、特色发展的教育理念，全力提高教育质量。积极发展学前教育，均衡优质发展基础教育，集约发展高中阶段教育，全面实施素质教育。发挥高校对中山发展的智力支持作用，办好省级职业教育基地，创新技能人才培养模式。促进民办教育健康发展。完善全民终身教育体系。做好教育体制改革试点工作。

迎接全国文明城市复检。大力弘扬“博爱、创新、包容、和谐”新时期中山人精神，激发市民文化自觉与文化自信。加强城市文明管理，开展文明镇区、文明社区、文明户标兵创建活动，积极变被动为主动，增创城市文明佳绩，确保蝉联全国文明城市。

发展公共卫生事业。深化医药卫生体制改革，稳妥推行基本药物制度，探索公立医院改革试点，鼓励社会资本举办医疗卫生机构。推进基本公共卫生服务均等化，加强基层医疗卫生体系建设，提供价格合理、便民质优的医疗卫生服务。完善疾病防控、药物保障和公共卫生应急体系，优化群众饮食用药安全环境。加快区域卫生信息化。

推进人口计生、体育等工作。科学调控人口规模，提高人口质量。坚持计划生育国策，落实人口计生层级动态管理和利益导向机制。实施体育惠民，扩大群众性体育设施覆盖面，提升竞技体育水平。发挥外事侨务、港澳事务、台务、民族宗教工作服务经济社会发展的重要作用，做好国防建设、双拥、统计、妇女儿童、人防、气象、地震、档案、方志等工作。

九、加快文化名城建设，提升城市文化实力

大力弘扬孙中山文化。把握纪念辛亥革命百年际遇，加快伟人故里建设，凝聚海内外乡亲智慧和力量，打造海峡两岸和世界华人共同精神家园。加强孙中山文化研究，办好孙中山文化节、“中山杯”华侨华人文学奖、海峡两岸中山论坛、逸仙奖等系列活动，推进孙中山文化、历史文化、产业文化、民俗文化、公共文化、博爱文化、“三名”文化、生态文化等“八大文化工程”，建设孙中山故居5A级国家旅游景区。发挥孙中山文化凝聚乡情、促进发展、展示城市形象的重要作用。

提升城市文化品质。发挥文化引领作用，实施城乡美化亮化净化艺术化“四化”工程。建设国家历史文化名城，加快建设新图书馆、博物馆群、画馆、非物质文化遗产展览馆、档案信息中心等一批重点文化项目，加强重点文物古迹和非物质文化遗产保护利用，构建普惠型公共文化服务体系。

积极发展文化产业。发展游戏游艺、影视动漫、创意设计等产业，建设好港口镇国家游戏游艺产业基地、小榄镇国家文化产业示范基地、粤台两岸文化产业园等文化产业平台。推动产业集群走科技与文化融合升级道路，提升产品文化附加值。

十、加快服务型政府建设，全面提高执行效力

强化为民服务意识。优化政府组织结构和运行机制，促进政府服务规范有序、公开透明、便民高效。坚持问政于民、问需于民、问计于民，弘扬求真务实作风，切实解决群众反映强烈的突出问题。坚持凝聚民心、汇聚民智、集聚民力，做好新形势下群众工作。坚持各级领导联系重点项目和企业制度，认真解决实际困难，加大力度帮扶企业发展。

加强政府作风建设。营造风清气正、廉洁从政的干事创业氛围，以加强政府建设实际行动，提高人民群众的满意度和幸福感。推进反腐倡廉制度建设，规范权力运行，完善治腐惩防体系。牢固树立全局观念、责任意识和拼搏精神，倡导鼓励进取、宽容失败、勇于担当、开放包容的社会风气。提高公务员适应发展方式转变、推动科学发展的能力，建设高素质干部队伍。

坚持科学民主依法行政。用科学发展观统领政府工作，自觉接受人大依法监督和政协民主监督，提高议案建议提案办理成效。主动听取各民主党派、工商联、无党派人士和各人民团体意见，健全公众参与、专家论证、社会公示、风险评估、合法性审查和跟踪反馈等科学民主决策机制。严格依法履行职责，规范行政自由裁量权，加强行政复议工作。强化审计监督和行政监察，推进政务公开、村务公开和网络问政。开展“六五”普法教育，弘扬法治文化。加强基层组织建设，巩固扩大基层民主。

大力提高行政效能。出政策、促转型、抓落实。以时间倒逼进度，以目标倒逼责任，集中力量打好重点工作攻坚战，力促行政提速增效。深化行政审批制度改革，健全审批联动机制，完善重大项目“绿色通道”。健全督办机制，强化行政问责，坚决制止行政不作为和乱作为。坚持厉行节约，降低行政成本，确保行政运行经费“五个零增长”。

以大发展保障大民生，把转型升级成果转化为人民群众福祉。在此，我代表市人民政府承诺，借助纪念辛亥革命100周年活动形成倒逼机制，围绕人民群众衣食住行等基本需求和日益增长文化需要等方面急待解决的突出问题，今年集中力量办好十件民生实事。一是大力推进交通畅行。建设新能源汽车示范线路，人民医院、中医院门诊部、城北公交枢纽站投入使用，力争公交出行分担率年内达20%，中心城区公共停车场新增1500个停车位，建设智能交通系统二期工程，公交服务质量不断改善。二是进一步改善治安环境。完成社会治安视频监控系统二期工程，连同一期共5891个监控点基本覆盖城乡。集中整治重点治安地区和重点治安问题，完善“一圈五区九线”治安防控网络，提高人民群众安全感。三是综合整治水环境。整治300公里内河涌，重点整治白石涌、崩山涌、西河涌、羊角涌和茅湾涌、麻子涌、红旗河、前山河流域等。实施岐江河净化美化工程，推动岐江夜游建设，年内形成中心城区段5公里滨水景观，着手推进建设全流域亲水宜居长廊。完成全禄水厂蓄淡抗咸、三角福隆泵站工程，保障全市用水安全。四是抓好“菜篮子”工程。运用价格平抑基金扶持蔬菜大棚、冷藏设施和平价门店建设，稳定物价，保障供给，优化食品安全环境。五是打造市民生活绿色环境。建设总长67公里、今年完成首期28公里的市区自行车道，建设田心森林公园。实施城市改造“穿衣戴帽”工程。六是推进文化卫生服务。建设兴中园名人雕塑，完善文化站、农家书屋等设施，做好镇区数字电视整体转换工作。开展15岁以下人群补种乙肝疫苗、免费出生缺陷产前筛查、农村妇女住院分娩补助等工作，落实9项基本公共卫生项目。七是完善社会保险体系。在户籍居民全部纳入城乡医疗保险一体化基础上，将非本市户籍职工和大中专学生纳入门诊基本医疗保险。八是增加保障性住房供给。改善中低收入家庭住房条件，通过廉租房租金补贴、廉租房、经济适用房、公共租赁房途径，提供保障性住房2700套，完成570间低保家庭危房改造。九是加强社会救助。提高最低生活保障标准。在中央发放困难群众临时性价格补贴基础上，向全市2.8万城乡困难群众发放一次性生活补贴和临时性价格补贴。帮助4300多名低收入对象参加城镇基本医疗保险，为2万名75岁以上老人安装紧急呼叫装置。十是加大富农强农力度。落实粮食直补、农资综合补贴、农作物良种补贴、农机购置补贴等政策，提高超级稻、冬种马铃薯、玉米种植补贴额度。通过办好十件实事，让城市更和美，让人民更幸福。

各位代表，同志们！回顾过去，光辉的历程令人鼓舞；展望未来，发展的蓝图催人奋进。时代的召唤，是我们前进的不竭动力；人民的幸福，是我们奋斗的不懈追求。我们坚信，有市委的坚强领导，有市人大、市政协和社会各界的大力支持，有300万中山建设者的同心协力，有上百万海外乡亲的倾情相助，我们一定能够不辱使命、不负重托，一定能够圆满完成今年和“十二五”各项目标任务，一定能够在科学发展的新征程上，昂首阔步前进，铸就新的辉煌！
